# Assessment of geographical accessibility to COVID-19 testing facilities in Nepal (2021)

**DOI:** 10.1016/j.lansea.2024.100436

**Published:** 2024-07-01

**Authors:** Parvathy Krishnan Krishnakumari, Hannah Bakker, Nadia Lahrichi, Fannie L. Côté, Joaquim Gromicho, Arunkumar Govindakarnavar, Priya Jha, Saugat Shrestha, Rashmi Mulmi, Nirajan Bhusal, Deepesh Stapith, Runa Jha, Lilee Shrestha, Reuben Samuel, Dhamari Naidoo, Victor Del Rio Vilas

**Affiliations:** a Amsterdam Business School, University of Amsterdam, Amsterdam, The Netherlands; b Karlsruhe Institute of Technology, Karlsruhe, Germany; c Department of Mathematical and Industrial Engineering - Polytechnique Montréal, Canada; d ORTEC, Zoetermeer, the Netherlands; e World Health Organization Country Office for Nepal, Kathmandu, Nepal; f Principal Investigators, Project Lead; g National Public Health Laboratory, Kathmandu, Nepal; h World Health Organization South East Asia Regional Office, New Delhi, India; i UK Health Security Agency - Public Health Rapid Support Unit, UK

**Keywords:** Geospatial data analysis, Access to COVID-19 diagnostic laboratories, Optimised location of health facilities

## Abstract

**Background:**

Ensuring equitable physical access to SARS-CoV-2 testing has proven to be crucial for controlling the COVID-19 epidemic, especially in countries like Nepal with its challenging terrain. During the second wave of the pandemic in May 2021, there was immense pressure to expand the laboratory network in Nepal to ensure calibration of epidemic response. The expansion led to an increase in the number of testing facilities from 69 laboratories in May 2021 to 89 laboratories by November 2021. We assessed the equity of physical access to COVID-19 testing facilities in Nepal during 2021. Furthermore, we investigated the potential of mathematical optimisation in improving accessibility to COVID-19 testing facilities.

**Methods:**

Based on up-to-date publicly available data sets and on the COVID-19-related daily reports published by Nepal's Ministry of Health and Population from May 1 to November 15, 2021, we measured the disparities in geographical accessibility to COVID-19 testing across Nepal at a resolution of 1 km^2^. In addition, we proposed an optimisation model to prescribe the best possible locations to set up testing laboratories maximizing access, and tested its potential impact in Nepal.

**Findings:**

The analysis identified vulnerable districts where, despite ramping up efforts, physical accessibility to testing facilities remains low under two modes of travel—walking and motorized driving. Both geographical accessibility and its equality were better under the motorised mode compared with the walking mode. If motorised transportation were available to everyone, the population coverage within 60 min of any testing facility (public and private) would be close to threefold the coverage for pedestrians within the same hour: 61.4% motorised against 22.2% pedestrian access within the hour, considering the whole population of Nepal. Very low accessibility was found in most areas except those with private test centres concentrated in the capital city of Kathmandu. The hypothetical use of mathematical optimisation to select 20 laboratories to add to the original 69 could have improved access from the observed 61.4% offered by the laboratories operating in November to 71.4%, if those 20 could be chosen optimally from all existing healthcare facilities in Nepal. In mountainous terrain, accessibility is very low and could not be improved, even considering all existing healthcare facilities as potential testing locations.

**Interpretation:**

The findings related to geographical accessibility to COVID-19 testing facilities should provide valuable information for health-related planning in Nepal, especially in emergencies where data might be limited and decisions time-sensitive. The potential use of publicly available data and mathematical optimisation could be considered in the future.

**Funding:**

WHO Special Programme for Research and Training in Tropical Diseases (TDR).

**Research in context**

**Evidence before this study**

We searched Google Scholar, and Scopus databases using the keywords such as “Healthcare equity in pandemic response”, “COVID-19 testing accessibility”, “Geographical analysis of healthcare facilities”, and “optimisation of COVID-19 testing locations”. To the best of our knowledge, literature on the importance of equitable access to healthcare facilities has mainly focused on urban or densely populated regions, often at a lower resolution at different administrative levels such as counties or provinces. Strategies to optimally invest in public health infrastructure like COVID-19 testing laboratories did not explore the use of high-resolution globally available open datasets coupled with official country datasets and application program interfaces (APIs) for near-real-time travel time computations. Such an approach is especially relevant for regions with challenging and diverse terrain such as Nepal.

Previous studies on healthcare accessibility during the COVID-19 pandemic did not look at how socioeconomic factors influence access to testing facilities. To start addressing this gap, our study used the relative wealth index which is a globally available open dataset as a proxy indicator to understand disparities in access due to economic factors.

**Added value of this study**

This study uniquely contributes by employing publicly available global data sets coupled with publicly available official data sources from the country, to assess COVID-19 testing accessibility in Nepal considering the location of population households at a 1 km^2^ resolution and the estimated headcounts of those households. In addition, the study uses state-of-the-art realistic travel time calculations and includes both walking and motorized travel modes. The use of open data enhances the replicability and applicability of the findings in similar contexts globally. We employed a mathematical optimisation model, solved with Gurobi,^1^ to identify the optimal locations for additional testing laboratories in Nepal, maximising population coverage while considering the heterogeneity in relative wealth as a proxy indicator for transportation availability.

**Implications of all the available evidence**

Our study highlights the need for a more equitable distribution of COVID-19 testing facilities in Nepal. Our findings suggest that accessibility is particularly low in mountainous regions and areas with lower relative wealth. This highlights the importance of considering transportation availability and the heterogeneity of population density when planning for testing infrastructure. Our study also aligns with WHO guidelines for establishing a tiered laboratory network that ensures geographical coverage and equity in testing access. Using open globally available data and mathematical optimisation approaches facilitates replicability and provides a valuable tool for health-related planning not only in Nepal but also globally in resource- and time-constrained emergencies where data is often scarce.

## Introduction

The COVID-19 pandemic led countries to implement various response measures to protect their populations' health. Testing strategies monitor the spread of the disease in different regions which help inform interventions like promoting social distancing^2^^,^^3^ and travel restrictions. However, mobility restrictions can hinder access to testing if testing facilities are not widely available. In Nepal, as of November 2021, a total of 89 laboratories were operational, each serving only the population within its respective province. These laboratories were either government-owned or privately operated. An alternative to fixed testing facilities is the use of mobile laboratories which can take different forms.^4^ Mobile laboratories offer additional capacity and flexibility to meet varying testing demands, as they can be relocated based on the spread of the infection. However, during a prolonged incident like the COVID-19 pandemic, decisions regarding their deployment can be challenging. Moreover, the distribution of these facilities should contribute to a service network that aims to be accessible to a significant portion of the population. Since the concept of accessibility refers to the population's ability to reach goods or services, whether commercial (restaurants, shops, etc.) or social (education and medical services), there is no single, definitive definition of the concept.

The importance of ensuring equitable access to healthcare facilities, as well as the current situation in Nepal, has been assessed by Cao and colleagues.^5^ While their study focuses on the overall state of affairs, ours specifically examines the availability of SARS-CoV-2 testing laboratories during the response to the COVID-19 pandemic. Current literature including reviews related to^6^, ^7^, ^8^ accessibility measures state that future studies should consider the travel costs to the service location, as well as the number and distribution of opportunities, particularly spatial distribution. In particular, travel cost models suggest that the greater the time or distance required to reach a destination, the less accessible that destination becomes. Similarly, a previous case-study^9^ stated that important elements of an accessibility measure include the number of opportunities for clients to access a service, the costs associated with traveling to meet the demand, and a measure of spatial segregation, which aims to define an inclusive service network that avoids excluding regions within the potential service area (especially crucial in the case of pandemics). Therefore, several indicators can be defined to represent key characteristics of an accessibility measure.

In light of the above considerations, our study aimed to: (i) assess the geographical distribution of COVID-19 testing facilities in Nepal, (ii) define the accessibility of these laboratories, and (iii) utilise mathematical optimisation approaches to maximise population coverage, considering the heterogeneity in relative wealth as a proxy indicator for transportation availability to reach the laboratories. Furthermore, Nepal's challenging geography^10^ necessitates measuring accessibility by accounting for actual travel times. After a careful comparison, validated by colleagues in the field, we selected MapBox^11^ as the provider of accurate distances and travel times using the local road infrastructure while reflecting the geography and average traffic conditions to use for this study.

## Methods

### Data sources

The following data were used for the current study.

#### Google geocoding API (June 2021)

##### Google maps platform

Geocoding is the process of converting addresses (like “1600 Amphitheatre Parkway, Mountain View, CA”) into geographic coordinates (like latitude 37.423021 and longitude −122.083739), which you can use to place markers on a map or position the map. The Geocoding API^12^ provides a direct way to access these services via an HTTP request. We used this service via a python API to geocode the location of COVID-19 test laboratories in Nepal, which was then manually verified by our team. The use of open-source services geocoding such as those based on OSM proved insufficient for our needs due to lack of accuracy in Nepal.

#### MapBox isochrone API (July 2021)

##### MapBox isochrone API

The Mapbox Isochrone API^13^ computes areas that are reachable within a specified amount of time from a location and returns the reachable regions as contours of polygons or lines that you can display on a map. and allow to identify the households that meet the corresponding accessibility thresholds with the need to compute exact individual travel times.

#### Digital elevation models (April 2024)

##### Stamen terrain

Terrain maps, featuring hill shading, used as background tiles on the map figures.^14^ The shading serves as a digital elevation model to show the challenging geography of Nepal.

#### Beneficiaries/population from world pop (June 2021)

##### Population counts

Estimated total number of people per grid-cell. The dataset is available to download in Geotiff and ASCII XYZ format at a resolution of 30 arcseconds (approximately 1 km at the equator).^15^ The projection is Geographic Coordinate System, WGS84. The units are the number of people per pixel with country totals adjusted to match the corresponding official United Nations population estimates that have been prepared by the Population Division of the Department of Economic and Social Affairs of the United Nations Secretariat (2019 Revision of World Population Prospects). The mapping approach is Random Forest-based asymmetric redistribution.

#### Potential locations for laboratories (June 2021)

##### HealthSites.io

The repository is well described.^16^ This data lists 1682 health centers in Nepal.

#### Administrative boundaries (June 2021)

##### Nepal administrative boundaries

Nepal administrative level 0–2 and district (unnumbered) boundaries.^17^

#### COVID-19 laboratories (June 2021)

The Ministry of Health of Nepal published detailed descriptions of the laboratory facilities established in the seven provinces in response to the COVID-19 pandemic in PDF Files. The COVID-19 Dashboard of Ministry of Health, Nepal also published Situation Reports daily to give information on COVID-19 Cases, Deaths, Test Positivity Rate.^18^ These sites have been taken down after the pandemic, the data provided is now hosted by reliefweb.^19^

#### Relative wealth index (June 2021)

##### Nepal relative wealth index data from humanitarian data exchange

Researchers at the University of California–Berkeley and Facebook developed micro-estimates of wealth and poverty that cover the populated surface of all 135 low and middle-income countries (LMICs) at 2.4 km resolution. The estimates are built by applying machine learning algorithms to vast and heterogeneous data from satellites, mobile phone networks, topographic maps, as well as aggregated and de-identified connectivity data from Facebook. They train and calibrate the estimates using nationally representative household survey 20 data from 56 LMICs, then validate their accuracy using four independent sources of household survey data from 18 countries. They also provide confidence intervals for each micro-estimate to facilitate responsible downstream use.^20^

#### Other data sources

Before selecting the above data sources, many others were considered, in particular for road networks. We started with an extraction of roads from OpenStreetMap data made by WFP following UNSDI-T standards. The data is updated in near-real time from OSM servers and includes all the latest updates. Since this dataset does not include streets and pathways that have been published on a separate dataset (streets and pathways) we merged those. This dataset enables the computation of realistic travel distances with scalable algorithms such as contraction hierarchies. However, travel times lack realistic speed estimates, and we were forced to use MapBox instead. The travel times produced by MapBox were validated by local experts and considered accurate.

### COVID-19 laboratories

The data on COVID-19 laboratories and the date they became operational, as used in this study, were taken from the daily situation reports published by Nepal's Ministry of Health and Population (MoHP).^18^ These reports detail how Nepal's diagnostic network responded to the SARS-CoV-2 pandemic. Besides data on the numbers and distribution of cases and deaths across the country, the reports also provide details on the number of PCR tests each laboratory performed daily. For a discussion on the burden imposed by the pandemic on the different laboratories, including the frequency of visits and testing and the stress on the laboratories' capacity, we refer to the study by Bakker and colleagues.^21^ For the current study, we extracted the evolution of the diagnostic network in response to the pandemic activity as well as the contribution of individual laboratories to providing the population with diagnostic testing.

While MoHP provides situation reports since the beginning of the pandemic (January 28, 2020), we restricted our analysis to the period to May 1–Nov 15, 2021. The reason for this is twofold. First, for the considered period, we have consistent data reporting. Second, the period includes several operationally relevant phases about the pandemic activity and testing capacity development, e.g., in May and June 2021, the second wave of COVID-19 pandemic led to the biggest surge in infections up to this point, triggering yet another phase of establishing new laboratories across the country in response.

### Vulnerability of population

In this study, we utilised the relative wealth index data from Meta's Data for Good.^22^ The relative wealth index is a metric that predicts the relative standard of living within countries using privacy-protecting connectivity data, satellite imagery, and other innovative data sources. It is constructed by incorporating nontraditional data sources, such as satellite imagery and privacy-protected Facebook connectivity data. The index's accuracy is verified through comparison with ground truth measurements from the Demographic and Health Surveys. On a global scale, there are approximately 20 million micro-regions, each covering an area of 2.4 km^2^. The relative wealth index provides an estimation of the wealth level of individuals residing in each micro-region in relation to others within the same country.

In detail,^23^ the Relative Wealth Index is a statistical measure used to estimate the economic status or wealth of households within a population. It is derived from asset ownership and living conditions data, using methods such as principal component analysis (PCA) to aggregate multiple indicators into a single index. The index typically does not have a fixed scale; instead, it is often standardised around a mean of zero. Higher values indicate greater wealth, while lower values indicate poorer conditions. The scale and range can vary depending on the data and the specific method of calculation. Higher values, above zero, indicate households that are wealthier than the average. These households likely own more assets and have better living conditions. Lower values, below zero, represent households that are less wealthy than the average, with fewer assets and inferior living conditions.

The index allows comparisons across different geographic areas or demographic groups within a study, providing insights into the distribution of wealth and its impact on various outcomes like health, education, and access to services. It is particularly useful in environments where direct monetary measurements are difficult to obtain. This metric is instrumental for policymakers and researchers in identifying disadvantaged areas and populations, helping to tailor interventions and allocate resources more effectively.

### Facility catchment area and population covered

To ensure realistic travel times, our study took into account the country's existing infrastructure, such as roads and streets and whether these are for pedestrian and/or motorised access. We relied on reputable services that estimated travel times based on real-life data, and we validated their accuracy through consultation with local experts. After comparing Open Street Map, Google, Microsoft Here, and MapBox and confirming a selection of representative travel times with local knowledge, we selected MapBox as our preferred provider for the acknowledged accuracy in the field.

For calculating travel times within specified time frames, we utilised the isochrone API^24^ from MapBox.^11^ This API generates contour lines or vector representations of the reachable area for walking and driving modes. Our analysis included four isochrones: 30 min for both walking and driving, as well as 60 min for walking and driving.

Accessibility was measured by considering the average time required for walking and driving, utilising the local road infrastructure and a wealth of data. To assess population access, we established the four isochrones for each of the 89 existing laboratories, as described above. Each household was categorized accordingly to being part of those isochrones or not, enabling us to calculate coverage based on the headcounts corresponding to the households. Coverage represents the percentage of the population with access to at least one laboratory within the specified time threshold. We calculated coverage for two scenarios: (i) the actual scenario where testing was only allowed within the province of residence, and (ii) a scenario where movement between provinces was permitted.

### Optimisation model

Our optimisation model was based on the classical maximum coverage location problem by Church and ReVelle.^25^ In this problem, we specified the desired number of laboratories and assessed the number of households within the catchment area of each potential laboratory location. We assigned a weight to each household, reflecting its headcount. The model then selected locations in such a way that the combined population within their catchment areas had the highest weight, maximising the coverage (represented by the highest headcount of the population served within the joint catchment area). Such a model translated the decisions (which laboratories to open), the objective to meet (highest possible coverage of the population within the agreed metric), and the constraints (budget, i.e. a maximum number of laboratories, choose from suitable candidates, consider covered the households that are within the agreed metric of an open laboratory) into a mathematical formulation that a specific solver can handle. Once instanced with the relevant data, the resulting problem was solved to optimality, meaning the choice of laboratories that achieves the highest possible coverage. We used Gurobi^1^ to solve these so-called ‘mixed integer linear optimisation problems’ to optimality. In our case, the main decision is which subset of the potential sites to select for opening laboratories. To formalise the model, we used the following sets and parameters:

• *I*–the set of households, obtained as described in Beneficiaries/Population fromWorld Pop (June 2021). These are a total of 136093 individual points, as result of an aggregation to adjacent squares of 1 km^2^. Only the squares with a positive joint headcount are used.
• *J*–the set of potential health care facilities, obtained as described in Potential locations for laboratories (June 2021). These are 1682 locations in Nepal. For each of those, the four isochrones described in Facility catchment area and population covered were computed using MapBox^24^ as described in MapBox Isochrone API (July 2021).
• $J_{i}$–the set of potential health care facilities within reach of household i∈I. Note: $J_{i}\subseteq J$. This is obtained using the isochrones from MapBox Isochrone API (July 2021) in the following manner: if household *i* is inside the isochrone *j* for the metric considered, then $j\in J_{i}$.
• $w_{i}$–the weight of each household i∈I, its headcount. This is also from Beneficiaries/Population from World Pop (June 2021) and reflects the same aggregation as when defining *I*. It sums to 29,135,247 inhabitants on our dataset.

Each of the four metrics used leads to one instance of the problem, as the metric defines the sets $J_{i}$. The model revolves around binary decision variables $z_{i}$ for each household i∈I to indicate if that household can be served by a health care facility that is opened at j∈J, as indicated by the corresponding variable $x_{j}$. In that case, the household is covered and its headcount counts for the objective function value of the solution.

The complete model, as formulated below, states in the first line the objective as to maximize the total weight of the households served, while the second line (after subject to) lists the first constraint: each household is only served if at least one laboratory within reach is open. Then the number of laboratories to open is limited by *p*, a parameter that indicates the maximum number of locations to select.

$$\max\;\sum_{i\;\in \;I}w_{i}z_{i}$$

$$\text{subject}\;\text{to}:z_{i}\leq \sum_{j\;\in \;J_{i}}x_{j}\forall i\in I$$

$$\sum_{j\;\in \;J}x_{j}\leq p$$

$$x_{j}\in \left\{0,1\right\}\forall j\in J$$

$$z_{i}\in \left\{0,1\right\}\forall i\in I$$

The next subsections explain the two levels of decision-making that this mathematical optimisation model helps to address.

#### Pareto curves for strategic decisions

To address the decision of how much budget to allocate for the expansion, which translates into the number of new laboratories to open, one needs to balance the costs and the benefits. In our case, the costs relate to opening new laboratories and are bounded by the available budget, which leads to *p*. The benefits are the percentage of the population with access to at least one laboratory. Plotting the coverage obtained against the number of facilities added leads to a Pareto frontier that shows the optimal value of the optimisation problem defined above as a function of the parameter *p*, defining the budget to spend. To obtain such curves we need to solve the optimisation problem with increasing values of *p*. For that reason, unlike Church and ReVelle,^25^ we model the budget constraint as an inequality,^26^ while originally the model was proposed with equality. The reason is technical: the solution obtained for *p* remains feasible for p+1 enabling the solver to start with a valid solution in its search for a new optimum. These so-called ‘hot starts’ often decrease the time taken by the solver to find the optimal solutions.

#### Incremental optimisation for tactical deployment

It is important to note that the optimal solution to add, for example, ten additional facilities may not necessarily be a subset of the optimal solution to add 11 additional facilities. Therefore, this Pareto frontier does not indicate the specific facilities that will open over time. To define a good order for opening the laboratories, ensuring the highest gain at each step toward the optimal solution selected, we create a sequence of facilities to be added over time, leading to that optimal selection. For this purpose, we utilised a simple greedy algorithm applied to the sub-problem that consists only of the facilities in the optimal solution: at each step select the laboratory that yields the highest increment in coverage among all not yet opened laboratories in the optimal selection. This approach ensured effective and efficient implementation of facility additions over time.

### Role of the funding source

This work was funded by the UNICEF/UNDP/World Bank/WHO Special Programme for Research and Training in Tropical Diseases (TDR). The funding covered the use of commercial data services such as Google Maps and Map Box. Gurobi LLC gave access to a free license of the Gurobi optimisation Solver. The funders had no role in the design, data collection, analysis of the study, nor the decision to publish or prepare this manuscript.

## Results

**Fig. 1:**
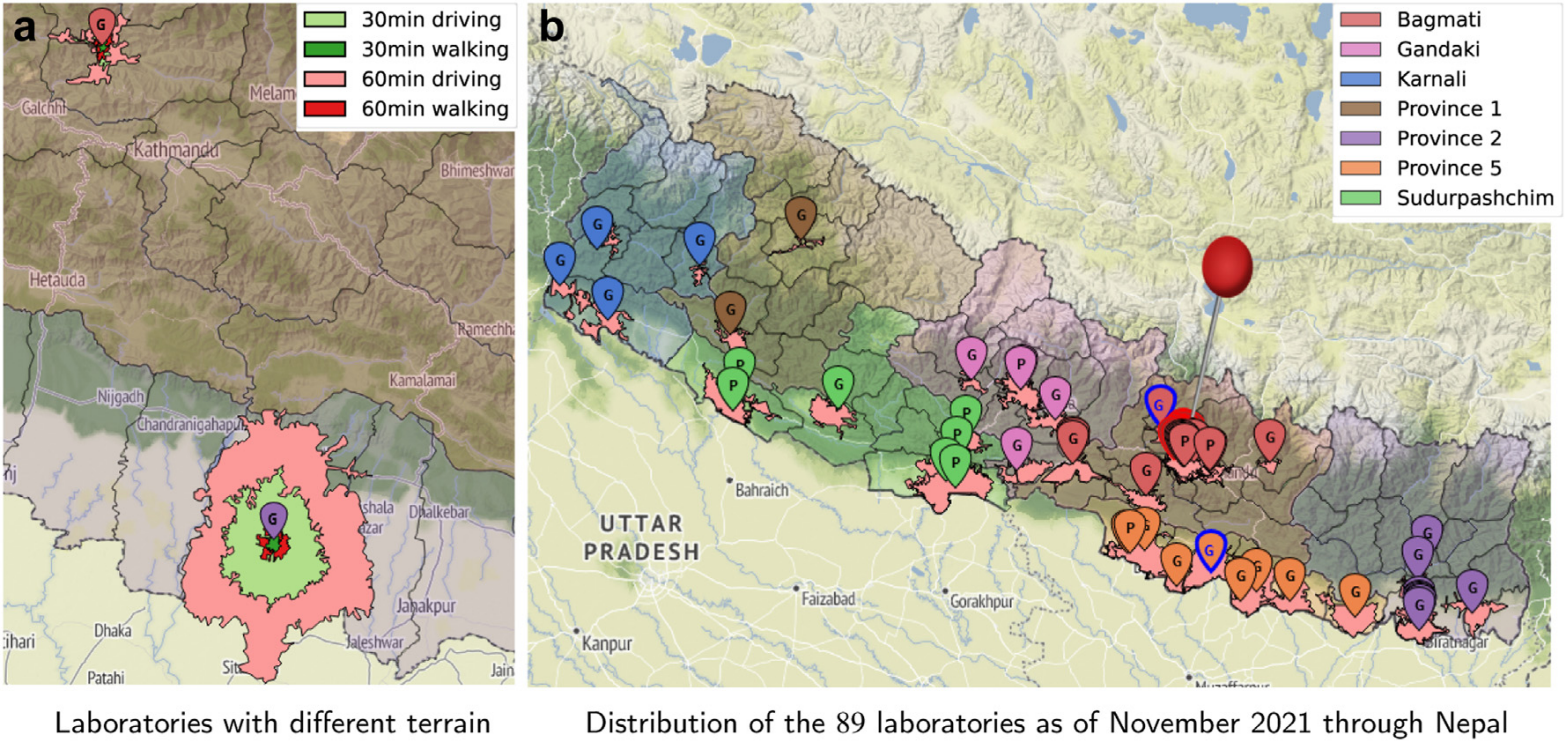
Subfigure b) on the right, the geographical distribution of the laboratories (Government and Private) as of November 2021, including the catchment areas of 1-h motorized traveling: the pale red polygons with a black boundary encompass the points within that travel time of the pin in their middle. Administrative areas and laboratory pins are coloured to identify the seven provinces of Nepal. On the left, in subfigure a), we zoom in on two laboratories and compare the catchment areas for the four metrics of travel times. These laboratories are identified with a blue border around their pins in b) and are situated at different elevations, clearly showing how geography affects travel times.

We used colour-coded figures to aid in presenting our results. We consistently use colours from two important sets, presented in Fig. 1: colours used to distinguish the access metrics and colours used to identify the country's province. Additional colours are used and explained for specific purposes.

### Descriptive analysis

Nepal has a very challenging geography, with elevations ranging from less than 100 m to over 8000 meters above sea level. A visual representation is provided in Fig. 1, including in b) a map with the laboratory locations on the whole of Nepal, color-coded according to the respective provinces. This map is shown on a background that depicts the mountainous characteristics of the ground, the differences in elevation being more frequent in the north than the south of Nepal. The letter, G or P, inside the pin locating each laboratory denotes a Government or Privately owned laboratory, respectively. Most of the laboratories are concentrated in and around the capital city of Kathmandu, identified by the taller pin. It should be noted that the areas for 1-h driving, while remaining in the same province as the laboratory is situated, are also depicted in b), clearly demonstrating that large portions of the Nepal surface do not have access to a laboratory within a 1-h drive. Only the population that resides inside such areas has the corresponding access. Fig. 1a) shows the two laboratories whose pin has a blue boundary in Fig. 1b). These were chosen to illustrate the impact of geography on accessibility. The top one, Trishuli Hospital in Bidur, northwest of Kathmandu, is at just 600 m above sea level, but on an already mountainous area, while the one on the bottom, Malangawa Hospital, southeast of Kathmandu, is at 100 m on a mostly flat area. Notice the respective areas of points within the four access metrics of traveling time to that laboratory. The area encompassed by the figure delimiting the 1-h drive is 104.4 km^2^ around Trishuli Hospital in Bidur, and 1843.8 km^2^ around the Malangawa Hospital. Note that to show more clearly the effect of geography, independently of the administrative constraints, the 1-h drive area is not delimited to the province or even country boundaries on Fig. 1a). We believe this visualisation helps in understanding the importance of accurate accessibility metrics, such as the ones adopted by this work.

#### Situation in November 2021

Of the 89 laboratories that were operational in November 2021, 30 were in Kathmandu (shown in Figure Fig. 1 with a red boundary around their pin). Observe that these pins overlap due to the small confinement on the nation's scale. While^21^ addresses the burden imposed by the pandemic on the different laboratories, including the frequency of visits and testing and the stress on the laboratories' capacity, we focus this work on the strategic decisions of where to locate laboratories based on the location and density of the population and the real-life accessibility efforts. These decisions are taken at an earlier moment, to enable installation and deployment. As of November 2021, the average number of laboratories per million inhabitants in Nepal was 3. Table 1 illustrates the distribution of laboratories per province, revealing noticeable heterogeneity. Bagmati province, for instance, offers more than 6 laboratories per million inhabitants, while Sudurpashchim province has only 1.

**Table 1:** Number of laboratories by province, their ownership, as well as the number of inhabitants and the number of laboratories per million inhabitants as of November 2021.

Province	Laboratories	Government owned	Privately owned	Inhabitants	Laboratories per million inhabitants
Bagmati	50	19	31	7,783,878	6.42
Gandaki	6	5	1	1,691,668	3.55
Karnali	2	2	0	1,078,543	1.85
Province 1	9	5	4	5,974,835	1.51
Province 2	9	8	1	5,648,457	1.59
Province 5	9	4	5	3,720,260	2.42
Sudurpashchim	4	4	0	3,237,606	1.24
Nepal	89	47	42	29,135,247	3.05

#### From May 2021 to November 2021

**Fig. 2:**
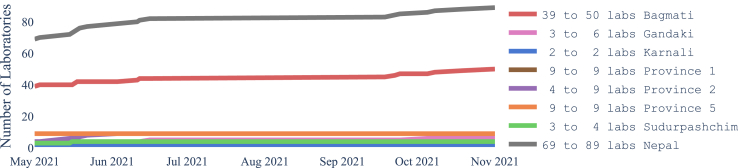
Number of COVID-19 laboratories in Nepal and per province from May 2021 to November 2021.

**Fig. 3:**
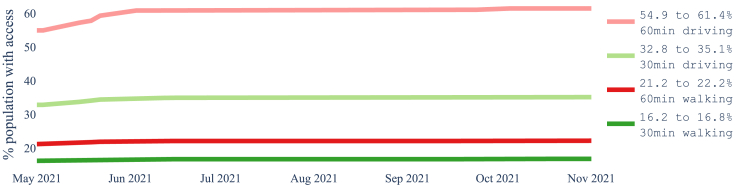
The proportion of the population with access to at least one laboratory within the stated threshold in the province of residence during May–November 2021.

**Fig. 4:**
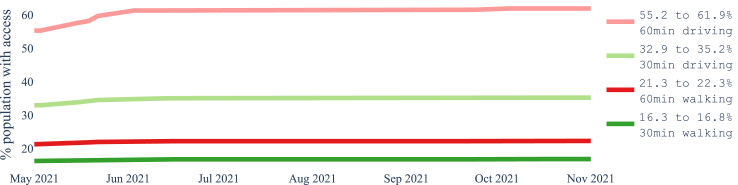
The proportion of the population with access to at least one laboratory within the stated threshold regardless the province of residence during May–November 2021.

Fig. 2 shows the increase over time in the number of laboratories from May to November 2021 in each province and the whole country, The increases are on the actual dates that laboratories were added. Figs. 3 and 4 show how the coverage, measured by each metric, increased in the whole of Nepal from May to November 2021. Fig. 3 reflects the case when access is restricted to the laboratories in the same province, while Fig. 4 shows a slight overall increase in accessibility if we remove the constraint of visiting laboratories within the province of residence. In particular, at the end of the period, 61.4% of the population had access to a testing laboratory within a motorized traveling time of 1 h while walking just 22.2% of the population can reach a laboratory within 1 h. Relaxing the ‘same province’ access restriction would have increased access both at the start of the period, in May 2021, and at the end, in November 2021.

**Fig. 5:**
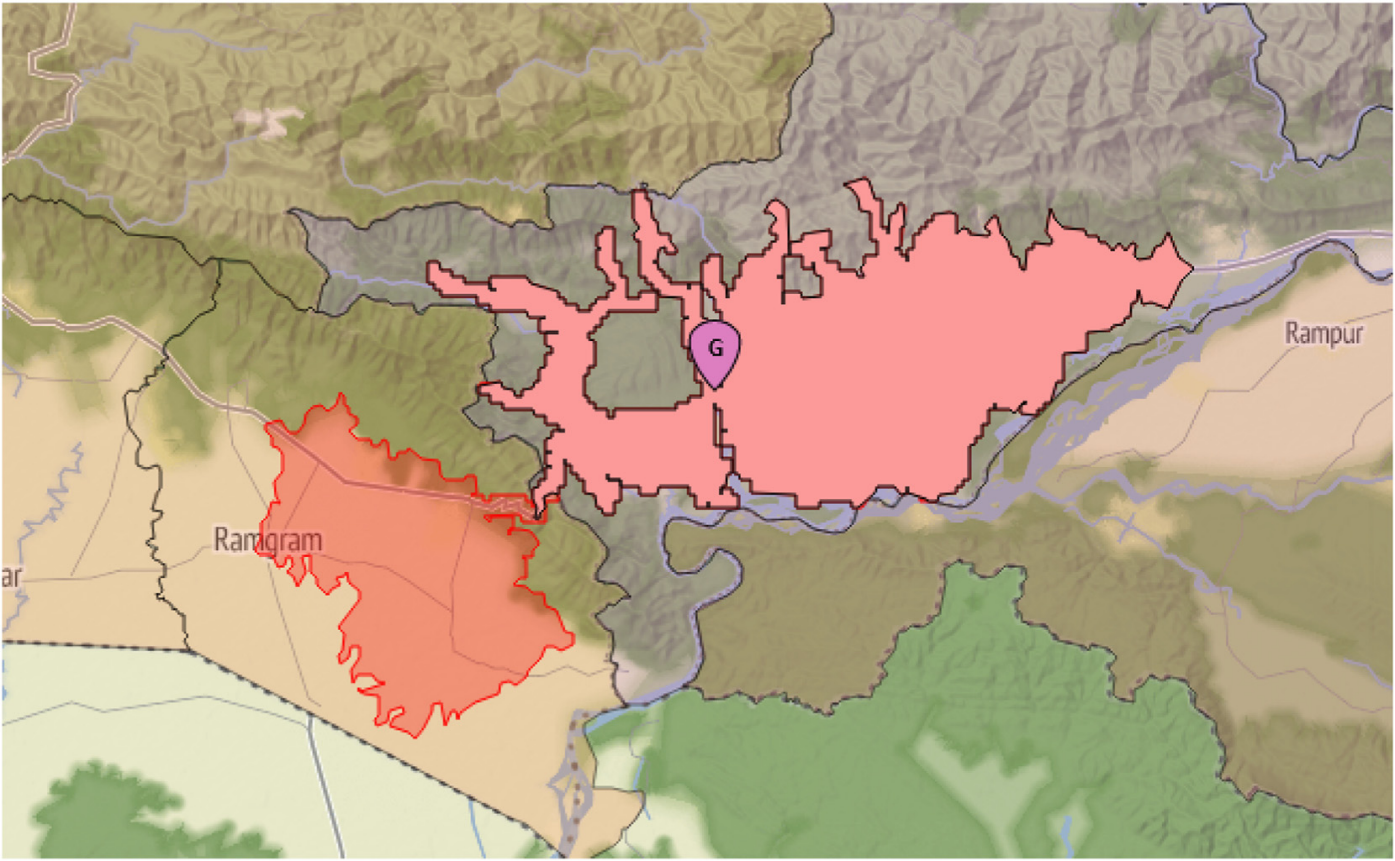
Madhyabindu Hospital in province Gandaki could serve additional 128,192 individuals within 60 min of driving time from Province 5, as shown by the left part, the translucid one, of the isochrone. Both parts are connected by the East-West Highway NH01.

The effect of relaxing the ‘same province’ constraint is the largest for laboratories close to province boundaries, see Fig. 5 for such an example. The changes are higher for the original 69 laboratories than for the final 89, meaning that, if at all possible, relaxing the administrative constraint would already have increased accessibility in May 2021.

### Effect of restricting access to the same province at district level

**Fig. 6:**
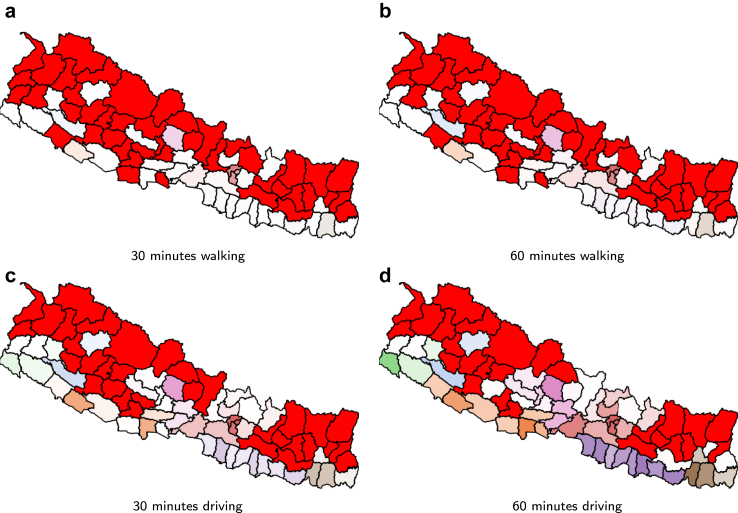
Accessibility per district to the closest, restricted, laboratory. The colours relate to the province color with darker colours indicating greater accessibility. Red is used to show the districts with no accessibility at all. The individual panels show the four accessibility metrics, as indicated in their own captions.

**Fig. 7:**
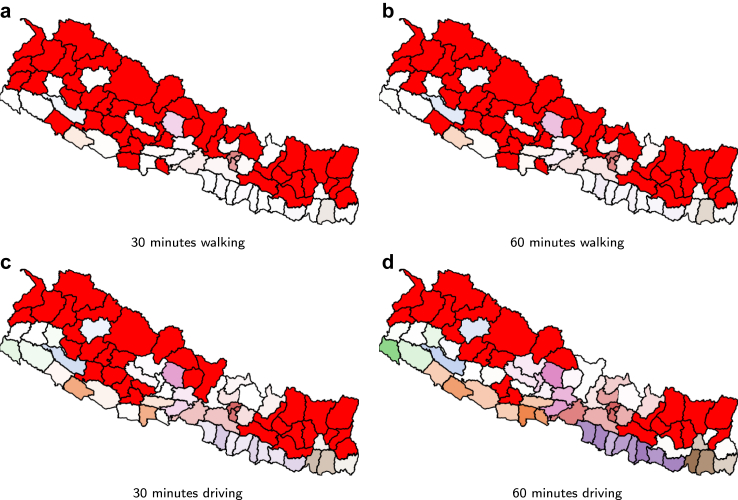
Accessibility per district to the closest, unrestricted, laboratory. The colours relate to the province color with darker colours indicating greater accessibility. Red is used to show the districts with no accessibility at all. The individual panels show the four accessibility metrics, as indicated in their own captions.

Considering the situation with respect to the 89 laboratories in operation from November 2021 as shown in Fig. 1, Figs. 6 and 7 show the accessibility by district for the two scenarios: restricted testing within the province, and not restricted. The latter returns a larger number of districts with access to at least 1 laboratory within the travel time thresholds. Tables 2 and 3 show a summary of the access in the same restricted and unrestricted cases. If only laboratories in the same province can be visited, and we consider 60 min of driving time then 26 out of the 77 Nepalese districts have no access at all. These districts are: Arghakhanchi, Baitadi, Bajhang, Bajura, Bhojpur, Dailekh, Darchula, Dolpa, Humla, Jajarkot, Kalikot, Khotang, Manang, Mugu, Mustang, Okhaldhunga, Panchthar, Pyuthan, Ramechhap, Rukum E, Rukum W, Salyan, Sankhuwasabha, Sindhuli, Solukhumbu and Taplejung. By relaxing the province constraint districts Dailekh and Salyan, both from the province Karnali, gain access, lowering the number to 24.

**Table 2:** Detailed coverage if only laboratories in the same province can be visited. Each sub-table per access metric lists 4 values per province in this order: the percentage of the population in the province with access to at least one laboratory, followed by the number of districts where no single person can access a laboratory and the minimum and maximum percentage of access per district in that province.

Province	60 min driving				30 min driving				60 min walking				30 min walking			
	%	#	min	max	%	#	min	max	%	#	min	max	%	#	min	max
Bagmati	82.69	2	2.07	100.00	74.98	2	0.01	96.50	67.60	5	3.52	91.00	56.35	5	1.00	78.10
Gandaki	40.30	2	0.44	89.80	21.67	4	0.03	71.60	11.82	7	0.69	48.10	7.97	7	0.29	34.00
Karnali	5.89	8	0.22	31.20	4.07	8	0.15	24.00	2.58	8	0.10	15.70	0.99	8	0.04	6.80
Province 1	59.41	7	0.45	87.60	28.08	10	5.76	41.80	6.39	10	1.31	24.60	2.32	10	0.40	14.10
Province 2	73.58	0	14.27	89.40	19.84	0	3.85	36.20	6.29	0	1.22	12.40	2.16	0	0.42	4.30
Province 5	50.73	3	0.45	99.10	23.96	5	0.48	69.30	5.29	8	0.67	31.90	2.30	8	0.29	16.10
Sudurpashchim	34.65	4	1.02	75.00	9.04	4	0.13	16.40	1.16	5	0.10	2.10	0.42	6	0.05	0.60
Nepal	61.43	26	0.44	100.00	35.11	33	0.01	96.50	22.18	43	0.10	91.00	16.79	44	0.29	78.10

**Table 3:** Detailed coverage if laboratories can be visited regardless of the province. Each sub-table per access metric lists 4 valuesper province and per accessibility metric, in this order: the percentage of the population in the province with access to atleast one laboratory, followed by the number of districts where no single person can access a laboratory and the minimumand maximum percentage of access per district in that province.

Province	60 min driving				30 min driving				60 min walking				30 min walking			
	%	#	min	max	%	#	min	max	%	#	min	max	%	#	min	max
Bagmati	82.69	2	2.07	100.00	74.98	2	0.01	96.50	67.60	5	3.52	91.00	56.35	5	1.00	78.10
Gandaki	43.64	2	0.69	89.80	23.32	4	0.03	71.60	13.14	7	0.76	48.10	8.67	7	0.29	34.00
Karnali	6.35	6	0.02	31.20	4.07	8	0.15	24.00	2.58	8	0.10	15.70	0.99	8	0.04	6.80
Province 1	59.64	7	0.45	88.00	28.08	10	5.76	41.80	6.39	10	1.31	24.60	2.32	10	0.40	14.10
Province 2	73.60	0	14.27	89.40	19.85	0	3.85	36.30	6.29	0	1.22	12.40	2.16	0	0.42	4.30
Province 5	52.27	3	0.45	99.10	24.00	5	1.12	69.30	5.29	8	0.67	31.90	2.30	8	0.29	16.10
Sudurpashchim	34.65	4	1.02	75.00	9.04	4	0.13	16.40	1.16	5	0.10	2.10	0.42	6	0.05	0.60
Nepal	61.88	24	0.02	100.00	35.21	33	0.01	96.50	22.25	43	0.10	91.00	16.83	44	0.29	78.10

Each sub-table per access metric lists 4 values per province and per accessibility metric, in this order: the percentage of the population in the province with access to at least one laboratory, followed by the number of districts where no single person can access a laboratory and the minimum and maximum percentage of access per district in that province.

### Equality

We extended the previous analysis considering the relative wealth index to inform equality in accessibility. We used micro-estimates of relative wealth at a 2.4-km resolution from^22^ to compute the median relative wealth at the district level. While we decided to consider households in the same district as having the same relative wealth in our work, other choices could be made, however since other choices would require validation, we opted for simplicity.

To visualise the situation we use scatter plots where the size of the dots reflects the population size of the district it represents, colored by the province it belongs to. Each dot is placed according to the median relative wealth index of the population in the corresponding district and the coverage: the percentage of the population within the district with access to at least 1 laboratory, either within or across provinces as indicated in each legend.

We add the linear trendlines corresponding to ordinary least squares to aid interpretation. The trendlines are computed within the provinces or for the whole country. Each trendline has the same color as the province and its districts or the color as used for Nepal in Fig. 2. Inequality shows in the form of clear positive trends between the proportion of population coverage and wealth, especially if benefiting mostly the higher relative wealth.

**Fig. 8:**
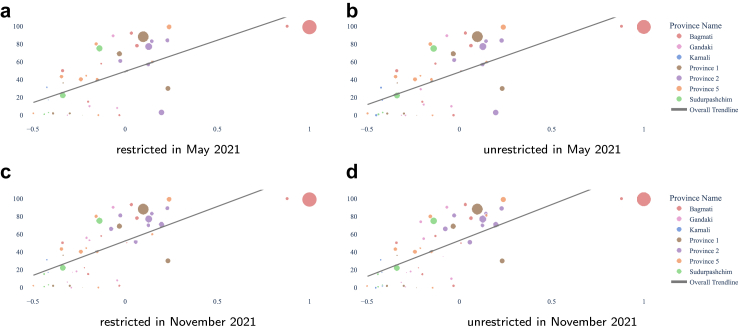
Accessibility per district to the closest laboratory within 60 min driving, restricted within the province of residence and not, at the beginning and the end of our period of study, as indicated by each panel's caption. For instance, a) depicts the situation as in May 2021 with the access restricted to laboratories cited in the province of residence. The vertical axis shows the percentage of access while the horizontal axis places the district bubble in the relative wealth index range. The size of the bubble is proportional to the number of inhabitants and the color reflects the province. The overall trendline shows that access grows with wealth. The individual panels show the four accessibility metrics, as indicated in their own captions.

**Fig. 9:**
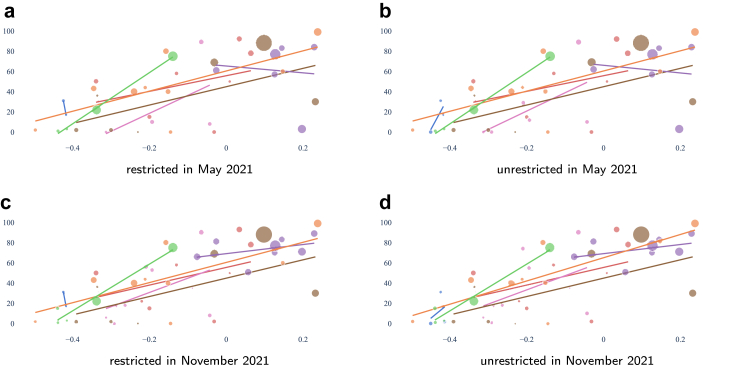
Accessibility per district to the closest laboratory within 60 min driving, without Kathmandu and Bhaktapur in the Bagmati province, under the same conditions as in Fig. 8. The trendlines are now per province. The individual panels show the four accessibility metrics, as indicated in their own captions.

We show only the figures for the most permissive metric that we consider: access within 60 min of driving. Fig. 8 shows the situation as of May 2021, when only 69 laboratories were in operation, and as of November 2021, with 89. It includes all districts, including two districts in Bagmati province with full coverage and the highest relative wealth, Kathmandu and Bhaktapur. Fig. 9 focuses on the less wealthy districts, omitting Kathmandu and Bhaktapur from the Bagmati province. In almost all cases we see that coverage grows alongside the growth of relative wealth.

### The potential of mathematical optimisation

The Joint External Evaluation (JEE) exercise conducted in Nepal in late 2022 identified operations research as one of the priority actions for the laboratory pillar.^27^ In order to illustrate the potential offered by mathematical optimisation, a fundamental tool of operations research, we described an optimisation exercise using data from HealthSites.io as described by Saamli and colleagues.^16^ This data lists 1682 health centers in Nepal that we take as possible laboratory locations. We do not imply that these are realistic choices, but we want to illustrate the potential of mathematical optimisation. An actual use of it would require careful identification of the candidate locations. We would have preferred using the apparently richer list of health centers used by Cao and colleagues^5^ but despite the description given in that paper, we could not find the data. That may explain why we observe lower accessibility as reported there. The difference may also lie in the metric used, but since the aforementioned paper does not offer the code used, we cannot compare.

### What if the 20 additional laboratories were chosen to optimise coverage?

**Fig. 10:**
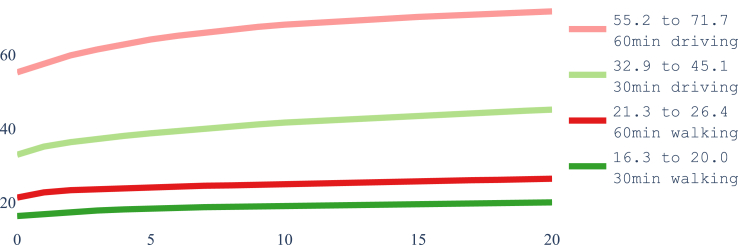
Pareto frontiers with the highest attainable coverage by optimal selection of 20 health centers to install as laboratories in addition to those in May 2021, selected from the 1682 health centers listed in Nepal by HealthSites.io plus the 20 actually used.

We now use the optimisation model presented in Optimisation model and show in Fig. 10 what could be attained if the 20 additional laboratories opened after May 2021 would have been selected optimally from all the potential laboratory locations listed by HealthSites.io plus the 20 ones that were actually opened. Since this section is illustrative of possibilities and no longer describes the situation in Nepal, we abandon the ‘same province’ rule. Redoing the analysis with the rule is straightforward, and we refer to^28^ where our code and data are freely available.

**Fig. 11:**
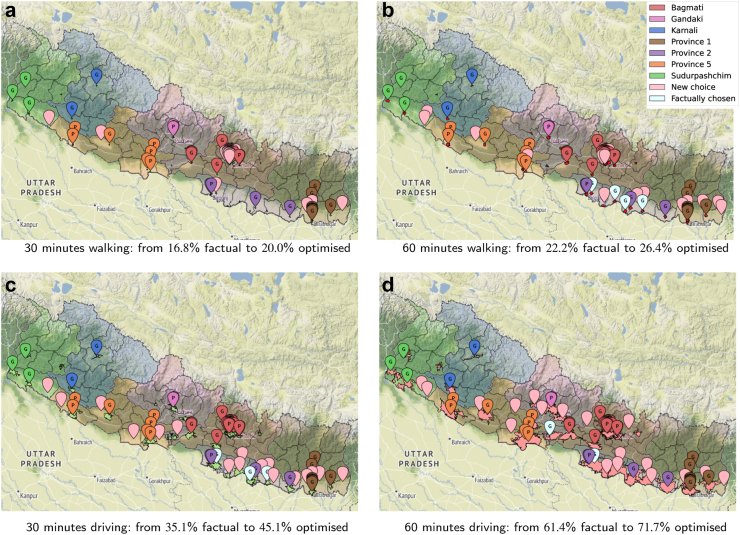
The optimal solutions with a budget for 20 laboratories in addition to the 69 in operation in May 2021 for each of the four different metrics considered, compared to the actual selection made in Nepal, when considering access regardless of province of residence. The solutions drawn correspond to Fig. 10 and the coverage is compared to the values in the factual solution as in Fig. 4. The pins of the locations selected in each case are colored to reflect if they match a location used in the factual solution implemented in Nepal as of November 2021 or are chosen from the new potential locations.

The four curves in Fig. 10 are Pareto frontiers. They tell a different story than in Fig. 3, which depicts the evolution in coverage for each additional laboratory, shown at the moment of its opening to the public. The horizontal axis of Fig. 3 is a timeline while that on Fig. 11 is a budget line. Each of the curves shows in Fig. 10 the highest attainable coverage for the corresponding accessibility metric with 1,2,3,…,20 additional laboratories. The end solutions, each obtained with 20 optimally chosen locations, are depicted in Fig. 11. This figure shows the four different optimal solutions, one per metric, with 20 additions identifying the laboratories that coincidentally where also opened in Nepal and those that are new. Each subfigure also depicts the isochrones of the corresponding metric. Furthermore, the legends of the four subfigures compare the coverage obtained by the corresponding solution (the highest attainable within the metric and a budget of 20) with the factual coverage within that metric as of November 2021. This is the potential offered by mathematical optimisation.

### Expansion

In this section we supposed that we could select the solution that maximises accessibility for 1 h of driving, as depicted in Fig. 11d), as the best one to implement. This selection entailed two decisions: the budget, 20 laboratories as in the expansion from May 2021 to November 2021, and the metric, to maximize the part of the population that has access within 1 h of motorized traveling time. To deploy this solution, we would need to open each of the 20 laboratories. One could then implement the sequence of 20 laboratory openings that at each step maximizes the gain in coverage. That can be done with a simple greedy selection from the pool of 20 optimal locations. We obtain a situation that evolves over time as described in Fig. 2, but instead for the laboratories selected by optimisation. After opening the last in the sequence, the coverage is the same as in the Pareto curve of the metric of choice, the 1-h driving, at the budget value of 20.

#### What are the limits?

**Fig. 12:**
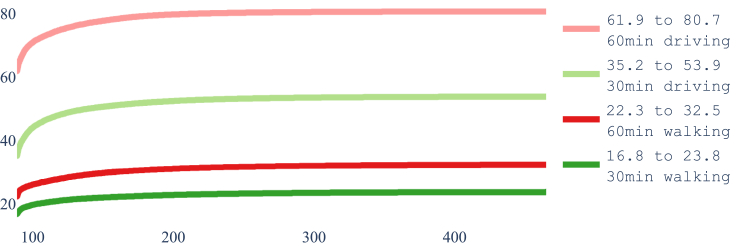
Highest attainable coverage by optimal selection with unbounded budget selected from the 1682 health centers listed in Nepal by HealthSites.io.

One additional question is: what would be the highest attainable coverage with an unlimited budget, and how many new facilities would it require to be reached? That question is answered by the Pareto frontiers depicted in Fig. 12. Two things are worth noticing:

1. In all cases just 375 out of the 1682 potential locations suffice to achieve the highest attainable coverage.
2. These values, which being optimal do not change even if we open all 1682 locations as laboratories, come quite short of the values reported by Cao and colleagues.^5^ As mentioned, this can be due to not having access to the same list of health centers and/or to a different implementation of accessibility metrics.

In any case, the discrepancies observed when comparing to the work by Cao and colleagues^5^ stress the value of both the data quality and the methodology adopted.

### Optimising with consideration for relative wealth

We will refer to the districts as ‘left’ and ‘right’ districts according to their positions on the scatterplots.

So far, our optimisation considered each individual as equal. In light of the differences in relative wealth, one may consider promoting accessibility for those who are relatively less wealthy. One way of doing so is to safeguard a minimum of laboratories to be opened in districts where we want to promote accessibility. This can be done as follows:

1. define a subset K⊆J of the potential laboratory locations as those in the districts that we want to promote.
2. decide on a minimum number *k* of choices to be taken from *K* in the optimal solution.
3. add the constraint $\sum_{j\in K}x_{j}\geq k$ to the optimisation model.

In our experiments, we took as *K* the subset of potential locations from left districts and we defined *k* as the optimisation budget *p* minus a number of locations allowed on districts in right districts. The case k=0 is the case when all laboratories (20 in our case) are allowed in any district and corresponds to optimising purely for headcounts, as in Fig. 10.

**Fig. 13:**
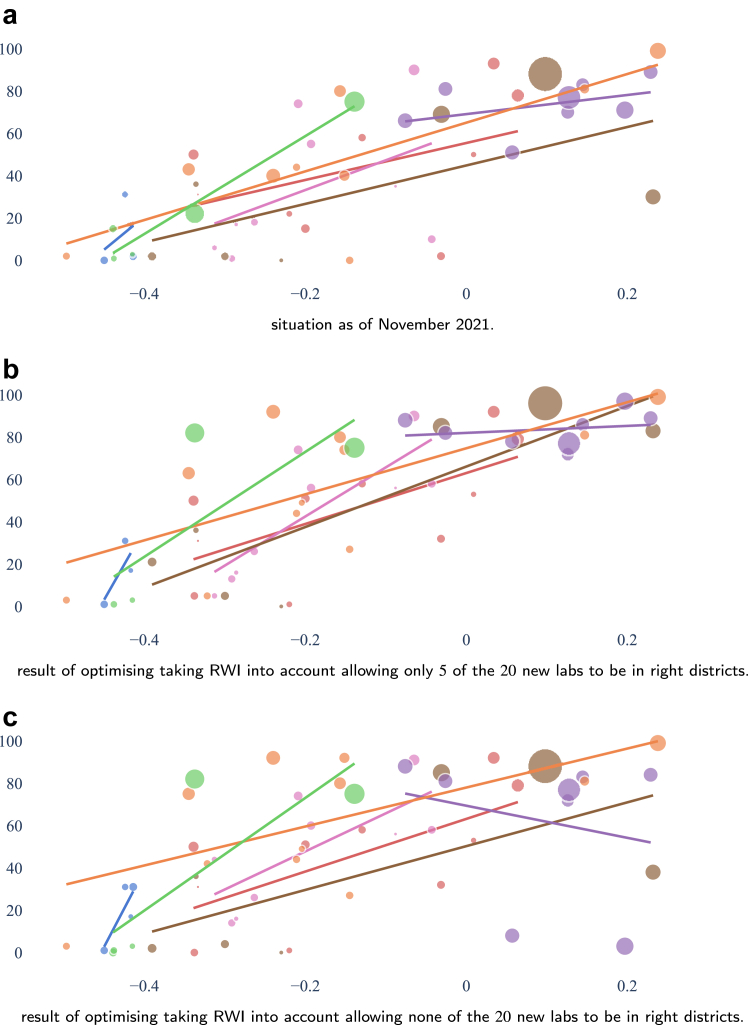
Accessibility per district to the closest laboratory within 60 min driving, as of November 2021 in a). Optimising taking relative wealth index into account by allowing only 5, shown in b), and none, shown in c), of the 20 new labs to be opened in right districts.

In Table 4, we summarised the outcomes of the situation as of November 2021 compared with the possible outcomes from the optimisation. Since allowing for any number above 13 repeated the solution obtained when allowing all, the table omits those rows. Notice that at a national scale, each situation depicted in Table 4 improves on the status quo as of November 2021 for all accessibility metrics considered. Our metric of choice, 1-h driving, attains its highest value when allowing only five laboratories to open in right districts. The last two columns show the percentage of the population with access within a 1-h drive, considering only the population in left or right districts. Note that 46.7% of the Nepalese reside in left districts while 53.3% reside in right districts. The best situation for left districts is, when the whole budget is reserved, no laboratories should be allowed in right districts. As soon as five laboratories are allowed in right districts we obtain the solution in Fig. 11d). Fig. 13 shows the effect of controlling the number of laboratories to open in left or right districts. We see clearly the effects on the accessibility across the relative wealth index scale.

**Table 4:** Comparison of the national coverage across the four different metrics plus a subdivision in left and right districts for the 1-h driving metric for the solutions obtained from the initial 69 laboratories as in May 2021 complemented with 20 laboratories chosen in sixteen different ways: factually as implemented in November, Fig. 4, allowing the given number of laboratories to open in right districts and the solution that maximises headcounts with access as in Fig. 10.

Situations	30 min walking	60 min walking	30 min driving	60 min driving	left	right
Fig. 4	16.8	22.2	35.1	61.4	35.2	85.3
Allow 0	17.4	23.6	39.9	66.3	50.4	80.2
Allow 1	18.0	25.0	41.9	68.4	50.4	84.2
Allow 2	18.5	25.5	42.5	69.7	50.1	86.9
Allow 3	18.9	25.6	43.1	70.5	49.6	88.8
Allow 4	19.2	25.8	43.7	71.2	49.1	90.5
Allow 5	19.4	25.9	44.0	71.7	48.6	91.9
Allow 6	19.5	26.0	44.3	71.7	48.6	91.9
Allow 7	19.6	26.1	44.6	71.7	48.6	91.9
Allow 8	19.7	26.2	44.8	71.7	48.6	91.9
Allow 9	19.8	26.2	44.9	71.7	48.6	91.9
Allow 10	19.8	26.3	45.1	71.7	48.6	91.9
Allow 11	19.9	26.3	45.1	71.7	48.6	91.9
Allow 12	19.9	26.4	45.1	71.7	48.6	91.9
Allow 13	20.0	26.4	45.1	71.7	48.6	91.9
Fig. 10	20.0	26.4	45.1	71.7	48.6	91.9

## Discussion

We aimed to assess the geographical accessibility to COVID-19 testing facilities in Nepal using different metrics: pedestrian and motorised travel. We also sought to illustrate the value of public data sources and mathematical optimisation in supporting decisions on expanding public health services to maximise population coverage.

Our results showed that the motorised mode delivers better geographical accessibility than the walking mode (see Fig. 3, where the proportion of the population served by the driving mode is several orders of magnitude larger than that by the walking mode). Unrestricted access to testing, regardless of province borders, revealed a larger number of districts with access to at least one laboratory, whether for pedestrian or motorised travel. Common across thresholds is the fact that valley districts in the south of Nepal have greater access to laboratories than districts in the mountainous areas of the north. We observed widespread heterogeneity in accessibility across districts, with wealthier districts having better accessibility to laboratories (Figures Figs. 8 and 9). These figures also show that smaller districts, in terms of population size, tend to have worse accessibility, although no formal analysis was conducted. Optimising the location of the 20 laboratories that opened during the period May–November 2021 would have led to improvements in accessibility across all thresholds. We illustrated that a simple way to benefit the population with lower relative wealth does not seem to compromise the nationwide results.

Nepal recently conducted its first Joint External Evaluation (JEE) exercise.^27^ The JEE report identifies several laboratory-specific strategic capacities for further development that demand subsequent operational and more granular planning. Our findings aim to contribute to this latter ask by supporting the Ministry of Health and Population (MoHP) decisions on the possible location of mobile laboratories for testing to service areas of greater need or vulnerability. We believe that wider recommendations could also be derived from our work. Mainly, the significance of conducting a needs assessment as done by the MoHP and WHO in late 2020 triggered this research. We would further suggest the deployment of mechanisms to capture this information regularly during outbreak response to populate panel analyses of capacities and capabilities. Our results could support government decisions on the possible location of mobile laboratories for COVID-19 testing and determining their service area. This is particularly relevant for future pandemics where rapid and equitable access to testing is crucial.

Our study is the first to assess healthcare accessibility, specifically screening for COVID-19, by geographical coverage of the population in Nepal. We utilised publicly available open data sources complementing the official data, and mathematical optimisation to inform strategic decisions. Our analysis demonstrated that optimising the location of testing facilities can improve overall accessibility, benefiting disadvantaged populations without compromising overall results.

Our work did not assess critical considerations for the effective readiness of testing laboratories, such as the availability and skillset of laboratory personnel to address sampling and testing demands. Such an exhaustive exercise would have required panel data on the capacities and capabilities of each operating laboratory, which was only available in the form of a cross-sectional survey conducted by the MoHP and WHO in November 2020. We did not explore nuances such as disease severity, comorbidities, and vaccination coverage, which may explain the observed testing behaviour and demand. Additionally, our models of coverage are not demand-driven as we did not consider variability in the number of tests required by the population as COVID-19 spread across the country. Furthermore, we did not consider population characteristics that might inform demand heterogeneity, such as some laboratories charging for testing while others offer free services. Using relative wealth for each district as a proxy indicator of transportation availability to reach laboratories presents several specific limitations. We could not validate the reliability of wealth as a measure of transportation accessibility due to the lack of data on car ownership and public transport availability. We only presented a crude association between relative wealth and accessibility without adjusting for potential confounders such as the proportion of elderly people. Additionally, we lacked granularity within districts to show different access levels, such as between rural and urban areas or between genders. We based our calculation of coverage on access to at least one laboratory within the established isochrones, not accounting for the capacity of the closest laboratory to handle more samples if demand increases. Pairing laboratories with others nearby might still provide equitable access to testing services, as reported by Bakker and colleagues.^21^

Our study highlights the significant disparities in geographical accessibility to COVID-19 testing facilities in Nepal and underscores the importance of strategic planning in public health services. By leveraging and combining different data sources and mathematical optimisation, we can inform better decision-making to enhance healthcare accessibility, particularly for disadvantaged populations, and we can do so even in the case of a future health emergency as the one observed during the SARS-CoV-2 pandemic.

## Contributors

Parvathy Krishnan Krishnakumari: conceptualisation, data collection, data verification, analysis, application of methodology, conclusions, manuscript production. Hannah Bakker: conceptualisation, data collection, data verification, analysis, methodology validation, manuscript review. Nadia Lahrichi: conceptualisation, data verification, analysis, methodology validation, manuscript review. Fannie L. Côté: conceptualisation, data verification, analysis, methodology validation, manuscript review. Joaquim Gromicho: conceptualisation, data verification, analysis, application of methodology, conclusions, manuscript production. Arunkumar Govindakarnavar: project lead, conceptualisation, manuscript review. Priya Jha: domain expertise, data validation, manuscript review. Saugat Shrestha: domain expertise, data validation, manuscript review. Rashmi Mulmi: domain expertise, data validation, manuscript review. Nirajan Bhusal: domain expertise, data validation, manuscript review. Deepesh Stapith: domain expertise, data validation, manuscript review. Runa Jha: domain expertise, data validation, manuscript review. Lilee Shrestha: domain expertise, data validation, manuscript review. Reuben Samuel: local and regional domain expertise, data validation, manuscript review. Dhamari Naidoo: local and regional domain expertise, data validation, manuscript review. Victor Del Rio Vilas: project lead, conceptualisation, manuscript review.

## Data sharing statement

All data used to produce this article, including code, is available.^28^

## Editor note

The Lancet Group takes a neutral position with respect to territorial claims in published maps and institutional affiliations.

## Declaration of interests

VJDRV was a WHO staff member at WHO SEARO during the conception and development of this study and is currently affiliated with The UK Public Health Rapid Support Team, which is funded by UK Aid from the Department of Health and Social Care and jointly run by the UK Health Security Agency and the London School of Hygiene & Tropical Medicine. VJDRV, PKK, NL, HB, JG, and FLC received funding from the WHO Special Programme for Research and Training in Tropical Diseases (TDR), provided to VJDRV via WHO SEARO, which covered the usage of some commercial geographical information systems. Additionally, PKK, NL, HB, JG, and FLC received funding from Gurobi LLC in the form of a Gurobi Optimisation Solver license. The views expressed in this publication are those of the author(s) and not necessarily those of the World Health Organization (WHO), the Ministry of Health and Population (MOHP) of Nepal, the Department of Health and Social Care of the UK, or any other affiliated organizations.
